# Detection of Diverse Sequence Types of Legionella pneumophila by Legiolert Enzymatic-Based Assay and the Development of a Long-Term Storage Protocol

**DOI:** 10.1128/spectrum.02118-22

**Published:** 2022-10-31

**Authors:** Sara Matthews, Hana Trigui, Marianne Grimard-Conea, Elliston Vallarino Reyes, Gabriel Villiard, Dominique Charron, Emilie Bédard, Sébastien Faucher, Michèle Prevost

**Affiliations:** a Department of Natural Resource Sciences, McGill University, Montréal, Québec, Canada; b Department of Civil, Geological and Mining Engineering, Polytechnique de Montréal, Montréal, Québec, Canada; c Centre de Recherche en Infectiologie Porcine et Avicole (CRIPA), Faculté de Médecine Vétérinaire, Université de Montréal, Montréal, Québec, Canada; d Industrial Chair on Drinking Water, Department of Civil, Geological and Mining Engineering, Polytechnique de Montréal, Montréal, Québec, Canada; University of California Davis

**Keywords:** sequence typing, isolation, genotyping, surveillance, storage, engineered water systems

## Abstract

Legiolert is a rapid culture-based enzymatic method for the detection and quantification of Legionella pneumophila in potable and nonpotable water samples. We aimed to assess the ability of this assay to detect diverse sequence types and validated a simple method to preserve samples. We used this assay on 253 potable and 165 nonpotable cooling tower water samples from various buildings in Québec, Canada, and performed sequence-based typing on 96 isolates. Six sequence types were identified, including ST1, ST378, ST1427, ST2859, ST3054, and ST3069. Whole-genome sequencing revealed that ST2859 was a member of the L. pneumophila subspecies fraseri. Additional tests with pure isolates also found that subspecies *Pascullei* and *Raphaeli* could be detected via Legiolert. Eight storage methods, including the current recommendation to store Legiolert trays at 4°C, were evaluated for their ability to preserve viable cultures. Of those, storage of Legiolert culture with 10% glycerol at −80°C produced the best results, fully preserving culturable *Legionella* for at least 12.5 months. We incorporated these findings into a standard procedure for processing Legiolert packets. Overall, Legiolert captures a variety of common and new STs in addition to important L. pneumophila subspecies and can be easily stored, which allows the conservation of a population of isolates for later characterization.

**IMPORTANCE** Legionnaires’ disease is caused by the bacterium Legionella pneumophila, which can be found in a variety of water systems. When outbreaks of Legionnaires’ disease occur, it is necessary to find the water systems transmitting the bacterium to humans. Access to historical isolates from water system samples is key for success in identifying sources but current regulations and isolation protocols mean very few isolates are obtained and stored long-term. We showed here that the Legiolert test could detect and produce isolates of a variety of L. pneumophila subspecies and types. In addition, the Legiolert test medium containing a representative population of isolates could be preserved for at least 12 months at −80°C with the addition of glycerol to the test medium. Therefore, we confirmed that the Legiolert method could be a useful tool for retrospective analysis of potential sources for an outbreak.

## INTRODUCTION

Legionellosis is an infection caused by the Gram-negative bacteria *Legionella* (1). This illness consists of Legionnaires disease (LD), severe pneumonia, and a milder flu-like disease called Pontiac fever (1). Most cases occur in elderly and immunocompromised individuals and are caused by Legionella pneumophila serogroup (SG) 1 specifically (1). The incidence of Legionellosis has been on the rise in recent years, with cases increasing by 6-fold in the US and more than 8-fold in Canada from 2000 to 2018. As such, it has become one of the most common causes of drinking water-associated infections (2, 3). This represents a severe impact on the quality of life of many individuals and a significant economic loss, with an estimated lifetime economic burden of $835 million in 2014 in the United States alone (4).

LD is primarily contracted via the inhalation of contaminated aerosols generated by engineered water systems (EWS), including building water systems, fountains, humidifiers, cooling towers (1), wastewater treatment plants (5), ice machines (6), and even automobile air-conditioning systems (5). Accurate discrimination of *Legionella* isolates is necessary to identify a cluster of cases and the source of infection (7). Traditionally, isolation of L. pneumophila from EWS is achieved by using selective culture plates (2). Presumptive *Legionella* spp. colonies from those plates are then confirmed as *Legionella* by their inability to grow without l-cysteine and by serogrouping or pulsed-field gel electrophoresis (2, 7). While these methods can identify general environmental sources, it has rarely been successful in pinpointing the exact source for community-acquired LD on their own (2). This is partial because SG1 is prevalent and phenotypically and genetically diverse, with some SG1 strains not causing disease (1). Genomic typing schemes such as sequence-based typing (SBT) have been more successful (2, 8–11). The SBT scheme of L. pneumophila isolates was developed by the European Working Group on Legionella Infections (EWGLI) and uses PCR amplicon sequencing of a combination of seven housekeeping and virulence genes to classify strains into over 3000 sequence types (STs) (12). It has been used for surveillance within the European Legionnaires Disease Surveillance Network (ELDSNet) (13), in the USA (11), Canada (14), and Japan (15) since 2010. As a result, infrastructure to support its use has rapidly developed, including a public database of STs (Public Health England, United Kingdom) and automated annotation programs for complete genomes like legsta (16). European Centre for Disease Prevention and Control (ECDC) and the Veterans Health Administration (6) have also prioritized supporting SBT as a first-line tool for outbreak investigation of *Legionella* spp (12).

Sequencing can be performed from DNA extracts from whole samples (e.g., water, biofilms, etc.) or isolates. Whole samples can contain more than one L. pneumophila strain, in which case STs can be impossible to determine as alleles cannot be associated with individual genomes. Therefore, it is preferable to use isolates. However, many factors can make isolation difficult such as interfering flora, low concentration of *Legionella*, and the fact that *Legionella* can enter a viable but non-culturable (VBNC) state or hide within a protozoan host (17–19). Some disease-causing *Legionella* species do not grow well using the traditional culture method (18). As a result, even clinical cases are difficult to confirm, with less than 13% being confirmed by culture in the EU (12). If SBT is to be utilized to its full potential, then culture-based detection of L. pneumophila will need to be improved.

The standard culture method is currently the gold standard for detecting L. pneumophila in both clinical and environmental samples. Briefly, a sample will be collected, diluted, then pretreated with acid, heat, or both before being plated on buffered charcoal-yeast extract (BCYE) supplemented with α-ketoglutaric acid and glycine, vancomycin, polymixin and cycloheximide (GVPC) (1, 20). Culture plates are incubated at 37°C with high humidity for 8 to 11 days. An atmosphere of 2 to 5% CO_2_ can also be used to further improve growth (1). Based on their morphology, suspected colonies then undergo further testing for verification of genus, usually by checking their ability to grow in the absence of l-cysteine, and serogroup and species, with antibodies or PCR. Therefore, this method is time-consuming, takes 10 days or more for full confirmation, is expensive, and requires expertise. Sensitivity is also highly variable due to the diverse attributes of samples, including the presence of interfering flora in nonpotable water especially, and the technicians' experience (1).

For environmental water samples, there is an alternative culture-based product that has been developed. Legiolert couples selective culture, bacterial enzyme detection technology, and the most probable number (MPN) method to quantify L. pneumophila in 7 days. MPN is a statistical method used to estimate the viable number of bacteria in a sample and is equivalent to the CFU that is traditionally reported by plating methods. It is based on two assumptions: bacteria are evenly distributed in broth, and inoculation with a single organism will result in a visible change due to growth (21). In the procedure, many replicates, or 96 wells in the Legiolert assay specifically, are inoculated with broth and incubated for a set period. Then, the number of replicates with visible growth is recorded, and the MPN that would produce that result is calculated. Compared to the standard culture method, Legiolert was found to have the same or better sensitivity and specificity for L. pneumophila (22, 23). As a result, interfering flora that would otherwise suppress *Legionella* growth is rarely an issue (<5%) (22–27), and positive results do not need further confirmation by PCR or antibody agglutination (28). Isolates can be obtained from positive wells by streaking their media on BCYE plates. Serogroups 1 and 2 to 14 have been detected with Legiolert and, in a comparative study by Barette et al., serogroups from Legiolert were the same as those isolated by standard culture more than 90% of the time (22, 23). As a result of its reliable performance, it was recently accepted as an American Society for testing and Materials (ASTM) standard (ASTM D8429-21) (29). Nevertheless, there are unanswered questions regarding Legiolert. First, it is unclear if Legiolert can capture diverse L. pneumophila subspecies and diverse STs. Second, long-term storage methods that maintain the viability of the bacteria have not been reported for Legiolert. Being able to revisit past samples is important in investigating the source of cases as there can be a lag between when an environmental source appeared and the emergence of an outbreak. During that time, the source could be disinfected, or changes in temperature or system operations could cause significant fluctuations in *Legionella* levels, making it difficult to isolate the strain from that source again. Finally, storage of samples is required in some jurisdictions. For example, in the province of Québec, Canada, it is required to preserve at least one isolate when *Legionella* levels exceed 10^6^ cells/mL in cooling towers (30).

In this study, we determined the ST of isolates obtained from the Legiolert assay using potable and nonpotable water samples from various infrastructures in Québec. The STs obtained were compared to those obtained from standard culture when available. We also developed a protocol to store positive Legiolert samples to preserve them for later strain identification in investigations.

## RESULTS

### Detection of L. pneumophila in collected water samples.

A total of 253 potable water samples from 2 locations and 165 nonpotable water samples from 17 locations were tested with Legiolert. *Legionella* was detected at both potable water locations and the sample positivity rate was high overall (76%). For nonpotable water, *Legionella* was only detected at 3 out of the 17 locations. The overall positivity rate of samples was 24% (Table 1). There were also 5 atypical results (3%) among nonpotable samples due to interfering flora. Those samples were deemed invalid. Atypical results occurred when trays had an abnormal appearance (ex. black or mold-containing wells), non-*Legionella* colonies grew on the GVPC culture plate, or colonies failed the l-cysteine test (Fig. S2 in Supplemental File 1). Of the 5 invalid results in this study, 2 were invalid because of the presence of mold in the well, 2 showed black wells, and 1 produced *Legionella-*like colonies on GVPC but grew on BCYE plates without cysteine.

**TABLE 1:** Summary of Legiolert results for potable and nonpotable water samples

Type of water samples	Total no. of locations sampled	Positive	Negative	Invalid	Total no. of samples
Potable	2	192	61	0	253
Nonpotable	17	39	121	5	165

The Association Française de Normalisation (AFNOR) NF T90-431 standard culture method was used in parallel on 37 nonpotable water samples. All results from the standard culture were valid as the expected results were obtained from the positive and negative controls. While interfering flora did appear on the direct plate of some samples, it did not invalidate the results. Often, the heat and acid treatment were effective in reducing or eliminating it. Paired sample results were congruent 30 times, with 23 negative and 7 positive tests (Table 2). Legiolert detected *Legionella* in another 2 samples, which appeared negative with standard culture, and had 5 invalid results. Of those, only 1 was positive in standard culture.

**TABLE 2:** Contingency table of results from paired Legiolert and standard culture on nonpotable water samples

Outcome	Legiolert		
	Positive	Negative	Invalid
Positive	7	0	1
Negative	2	23	4

### Sequence-based typing.

Sequence-based typing was performed on at least one colony from each test method for paired samples, and from 23 potable water samples that covered multiple sampling and time points for two buildings (Table 3). Overall, three STs were detected in 10 paired samples found positive by at least one of the methods. STs matched between the two methods for 6 of 7 doubly positive samples. In location S1, ST1 and ST3054, including partial allele profiles whose available genes (6 out of 7 genes) match ST3054, were found in roughly equal proportions within a single cooling tower system by Legiolert, whereas standard culture only detected ST1. Similarly in location S5, the standard culture method also only detected ST1 while Legiolert detected ST1 and ST3069, albeit the latter only once, indicating the dominance of ST1 in that system. Location S6, for which both potable and nonpotable systems were sampled, had similarly low diversity, with only ST378 being detected at 12 distinct sampling points in its potable water system. ST1 was found in the nonpotable system of this location, a cooling tower in this case. Lastly, ST1427 and ST2859 were detected at location S8, with both being found within the same Legiolert tray on two separate occasions. Taxonomic classification using MiGA found that ST1427 belongs to the subspecies *pneumophila* while ST2859 belongs to the subspecies *fraseri*.

**TABLE 3:** ST isolated from Legiolert and, if available, from standard culture by location, sampling point, and date^*a*^

Location	Sampling point	Sampling date	Sequence-based typing			
			Standard culture		Legiolert	
			No. of isolates typed	ST or allele profile	No. of isolates typed	ST or allele profile
Nonpotable						
S1	A B C D E F G H	2021-04-17 2021-05-27 2021-05-27 2021-05-27 2021-05-27 2021-06-01 2021-07-14 2021-07-14	ND^*a*^ ND ND ND ND ND 0 2	NA^*b*^ NA NA NA NA NA NA ST1	1 1 1 1 1 1 2 2	3_10_1_6_1_9_X^*c*^ ST1 ST1 3_10_1_6_1_9_X^*c*^ 3_10_1_6_1_9_X^*c*^ ST1 ST3054 ST3054
S5	A B B C D E B	2021-07-28 2021-07-28 2021-08-10 2021-08-10 2021-08-10 2021-08-10 2021-09-08	1 1 1 1 1 1 0	ST1 ST1 ST1 ST1 ST1 ST1 NA	1 1 1 1 1 1 1	ST1 ST1 ST1 ST1 ST1 ST1 ST3069
S6	A	2021-09-09	0	NA	1	ST1
Potable						
S6	A B C D E F G A B D E F H I J K L A	2021-07-20 2021-07-20 2021-07-20 2021-07-20 2021-07-20 2021-07-20 2021-07-20 2021-08-03 2021-08-03 2021-08-03 2021-08-03 2021-08-03 2021-08-03 2021-08-03 2021-08-03 2021-08-03 2021-08-03 2021-08-27	ND ND ND 0 0 0 0 0 0 0 0 0 0 0 0 0 0 0	NA NA NA NA NA NA NA NA NA NA NA NA NA NA NA NA NA NA	4 1 3 1 1 1 1 1 1 1 1 1 1 1 1 1 1 1	ST378 ST378 ST378 ST378 ST378 ST378 ST378 ST378 ST378 ST378 ST378 ST378 ST378 ST378 ST378 ST378 ST378 ST378
S8	A B C D E	2020-11-16 2020-11-16 2020-11-16 2020-11-30 2020-11-30	0 0 0 0 0	NA NA NA NA NA	2, 20 6 11 4, 4 1	ST1427^*d*^, ST2859^*d*^ ST1427^*d*^ ST1427^*d*^ ST1427^*d*^, ST2859^*d*^ ST2859^*d*^

a ST and allelic profiles were determined using Sanger sequencing of PCR amplicons or whole-genome sequencing. For partial ST profile, alleles are listed in the following order: flaA_pilE_asd_mip_mompS_proA_neuA. ND, standard culture was not performed for this sample.

b NA, not available.

c X, gene not detected.

d ST and allelic profiles determined by whole-genome sequencing.

### Investigation of interwell variability with whole genome sequencing.

**FIG 1:**
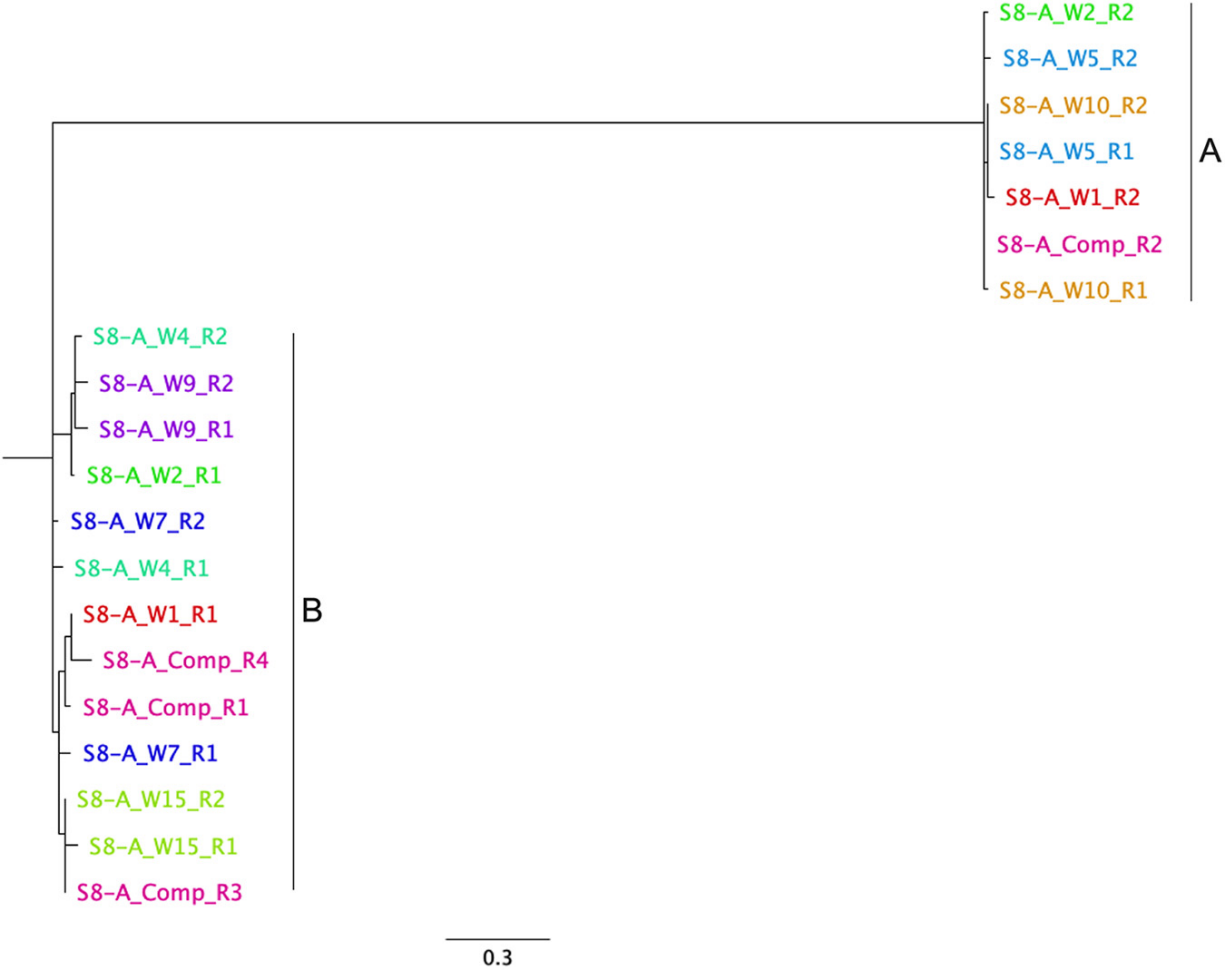
Parsimony tree of ST2859 isolates from Legiolert tray inoculated with sample S8-A. Genomes clustered in two distinct phylogenetic groups. Core SNP analysis was performed using kSNP version 3.021. The isolates are named and colored according to the well they were recovered from (W for well or comp for composite) and the replicate (R1 or R2). The scale bar indicates the changes per number of SNPs.

Whole-genome sequencing was performed on 2 colonies from 9 individual wells, and 4 colonies from a composite sample from the same Legiolert tray, S8-A (Table 3). ST1427 was found in one large well while all the remaining colonies were ST2859, representing 91% of all isolates sequenced (Table 4). We further analyzed the phylogenetic relationship, based on core genome single nucleotide polymorphisms (SNPs), between the ST2859 isolates recovered from this tray. Two ST2859 isolates were recovered from each of the 8 individual wells, and 4 isolates from the composite. As can be seen in Fig. 1, the isolates cluster in two groups diverging by 44 SNPs. For the small wells 5, 7, 9, 10, and 15 and the large well 4, their two isolates cluster together in the same phylogenetic group. For the large wells 1 and 2, one isolated cluster with group A while the other clusters with group B. For the composite, three of the isolates clustered with group B while one clustered with group A. These results suggested that Legiolert could capture the diversity of L. pneumophila within a system as different variants of the same STs can be recovered. It also indicated that the composite was sufficient to recover the different variants present in the wells of the Legiolert tray and by extension in the system being tested.

**TABLE 4:** Interwell variability of ST type in Legiolert tray inoculated with sample S8-A based on whole-genome sequencing of isolates^*a*^

Well number	Well size	No. of isolates typed	ST
1	Large	2	2859
2	Large	2	2859
3	Large	2	1427
4	Large	2	2859
5	Small	2	2859
7	Small	2	2859
9	Small	2	2859
10	Small	2	2859
15	Small	2	2859
Composite	NA	4	2859

a Isolates originating from the same well or composite all had the same ST type.

### Detection of other Legionella subspecies by Legiolert.

The isolation of subspecies *fraseri* from samples prompted us to test the performance of Legiolert in detecting the other subspecies of L. pneumophila. Fraquil suspensions of subspecies *Pascullei* and *Raphaeli* were inoculated in the Legiolert tray at a concentration of 5 × 10^3^ and 4 × 10^4^ cells/L, respectively. Both were readily detected by Legiolert with MPN of 2396 and 3614 cells/L, respectively, and both could be isolated again from their respective trays.

### Storage tests.

**FIG 2:**
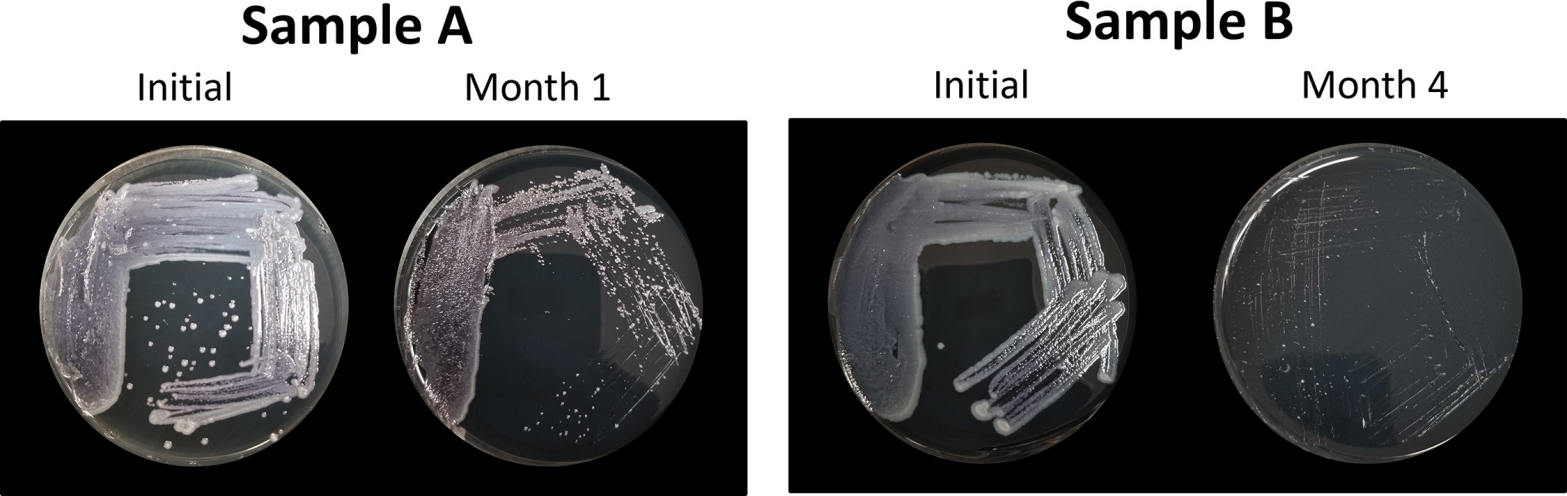
Rapid drop in viable L. pneumophila when Legiolert medium is taken from trays stored at 4°C after 4 months. Each Legiolert used in storage tests only had 2 large positive wells, so different samples were tested at each time point and compared to their corresponding initial plate.

**FIG 3:**
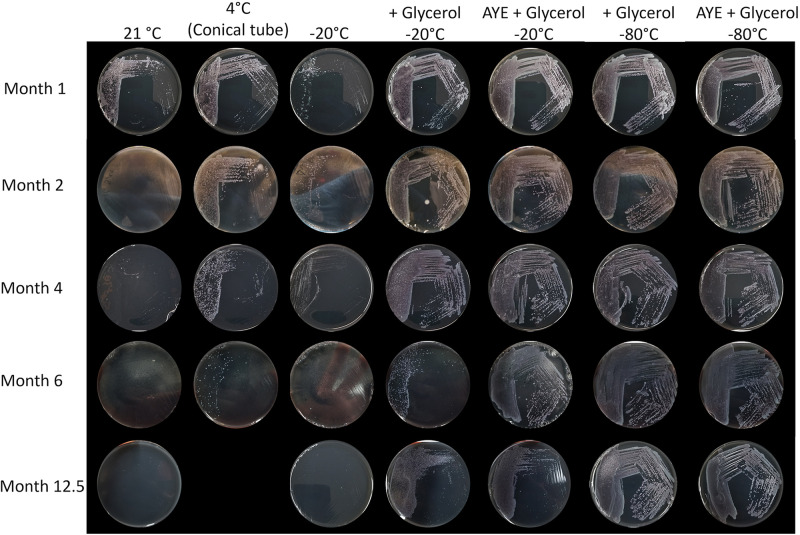
Recovery of Legionella from Legiolert trays stored by a different method. GVPC plates were inoculated with 100 μL of Legiolert sample A stored at room temperature (21°C), 4°C, −20°C, with 10% glycerol at −20°C or −80°C, or pelleted and resuspended in 10% glycerol in AYE at −20°C or −80°C.

**FIG 4:**
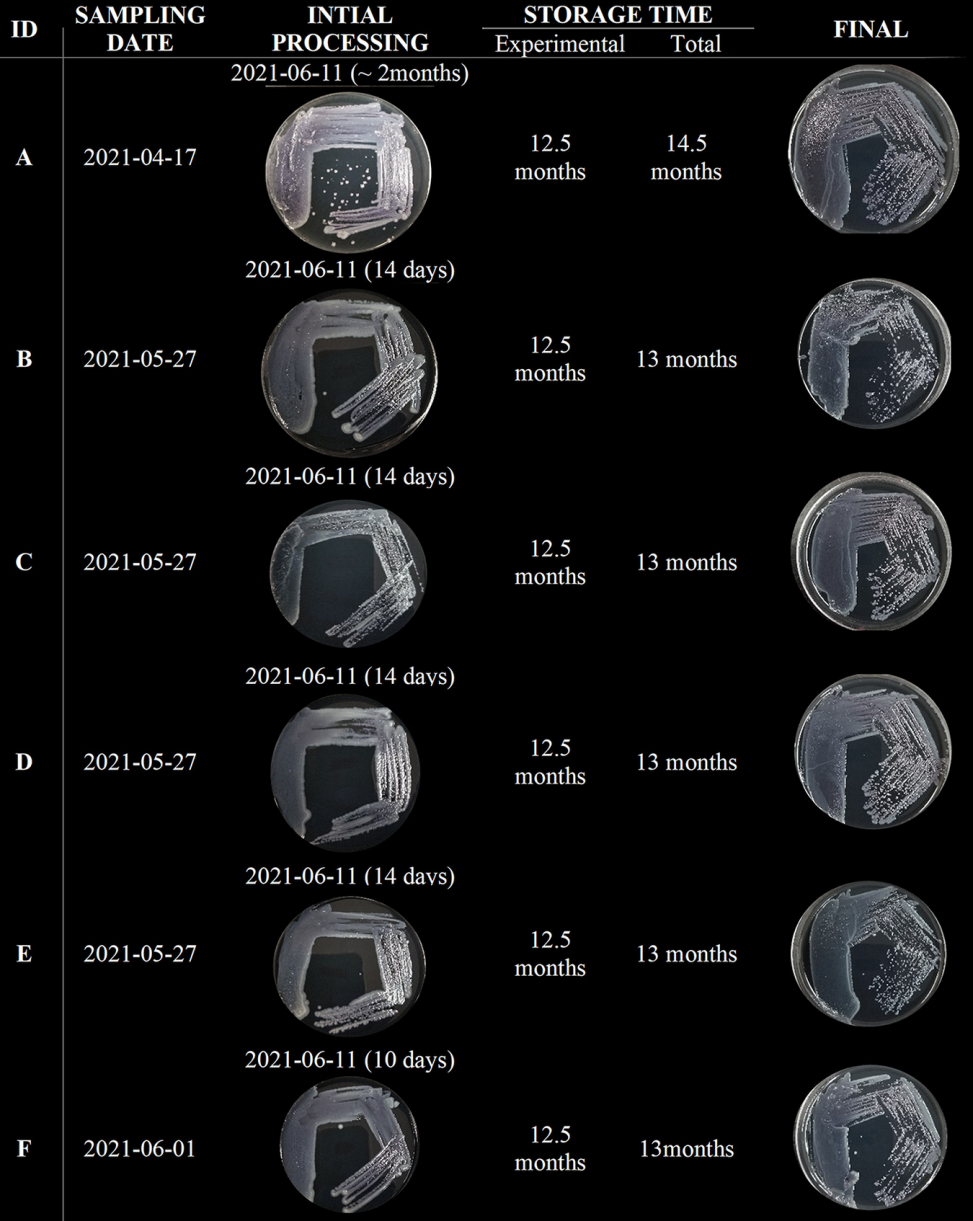
The final results of the recommended storage method, for which 10% glycerol was added to Legiolert media and stored at −80°C. The culture at initial processing and after 12.5 months was compared.

On the same day, medium from one large well of 6 different trays inoculated with samples from location S1, designated A to F in Table 3 with at least two positive wells, were cultured onto GVPC. The plates roughly showed the same amount of growth at this initial processing (Fig. 2 and 4). The remaining medium from the large wells was aliquoted into 100 μL and subjected to various storage conditions to determine, which maintained viable and culturable bacteria for at least 12 months. The trays, with one positive well intact, were kept and stored at 4°C. At each time point, one aliquoted sample from each tray and condition, and one tray with a large well still intact, were plated. When trays were stored at 4°C, bacteria were culturable for up to 4 months (Fig. 2). After 6 months in tubes at 4°C, only 2/6 replicates showed growth (Fig. 3). Unfortunately, a refrigerator malfunction prevented us from testing the 12-month time point for tubes kept at 4°C. Culturable bacteria were also lost after 2 months when the medium was stored at room temperature (21°C) and after 4 months at −20°C. The addition of 10% glycerol in Legiolert medium vastly improved preservation at −20°C, increasing storage time to at least 12.5 months for 4/6 replicates (Fig. 3). Decreasing storage temperature to −80°C preserved all samples completely for the entire experiment (Fig. 4). Samples stored in ACES-buffered yeast extract (AYE) broth with 10% glycerol at −20°C showed reduced growth compared to sample stored at −80°C grew after 12.5 months (Fig. 3).

## DISCUSSION

SBT and whole-genome sequencing (WGS) are essential tools for identifying the sources of sporadic cases and outbreaks. For them to be fully utilized, these techniques require isolates that standard culture methods can provide, albeit after an extensive and expensive 2 to 3 weeks process. Legiolert is a streamlined and rapid culture-based technique that provides results in 7 days and from which L. pneumophila can be cultured in 3 days. Our preliminary tests found that an abundance of colonies can be obtained easily by streaking Legiolert media on GVPC or BCYE. In this study, of 418 potable and nonpotable water samples analyzed with Legiolert, 231 were positive and only 5 were invalid (interfering flora), representing ~1% of all tests, or 3% of nonpotable which was consistent with previous studies (22, 23). These percentages for the Legiolert method correlate with several peer-reviewed publications (25–27). According to International Organization for Standardization (ISO) 13843 performance criteria, Legiolert was reported to have a specificity of >99% and spread plate culture (ISO 11731:2017), which is related to the AFNOR method, was reported to have a specificity is 95.3% (20, 31, 32).

In this study, SBT was performed on a total of 96 isolates. Of those, 71 isolates were from 23 positive potable water sites tested with Legiolert, 17 isolates from 14 positive nonpotable sites, and another 8 isolates from corresponding paired standard cultures (Table 3). Three STs were identified in potable water, including ST378, ST1427, and ST2859. ST378 and ST1427 have both been involved in clinical cases in Québec between 2005 and 2015 and all three have been previously found in the environment there (14, 33–35). In paired nonpotable samples, ST1, new ST3054, and ST3069 were identified. However, the latter two were only detected by Legiolert as standard culture was either negative or detected only ST1 in those cases. When both methods produced colonies, STs matched between them 6 out of 7 times, although again ST1 was the only type in congruent cases. This is likely due to the dominance of this strain at location S5 and the greater vulnerability of standard culture to interfering flora. This congruence between the methods is consistent with previous studies (22, 23). In a study comparing EnvironeX’s standard culture method and Legiolert, when both techniques detected L. pneumophila and serotype information was available, 18/20 showed detection of the same serogroup for 0.1 mL Legiolert, and 30/33 for 0.2 mL Legiolert (23). Rech et al. (22) found serotypes matched with the CDC’s standard method for all 27 cases in their study. Therefore, this rapid culture-based assay can capture both common clinically associated STs and new STs at a similar, if not greater, frequency to the standard method.

Legiolert trays consist of 96-wells and so individual wells can capture different strains. We combined an investigation of interwell ST variability with WGS to take an in-depth look at the genotypes of isolates from individual wells and a composite from a Legiolert tray inoculated with sample S8-A. Two isolates from one large well were ST1427. All isolates from the composite and 8 other individual wells, a total of 20 isolates, were ST2859 and MiGA classified them as *Legionella* subspecies *fraseri* (Table 4). This subspecies is clinically significant as it has been the cause of a large outbreak in Portugal in 2014 (9) and was the first strain associated with person-to-person transmission of LD (36). More recently there have been two reports of fatal coinfections with SARS-CoV-2 (37). We also confirmed that the other two subspecies of L. pneumophila can be detected using Legiolert.

**FIG 5:**
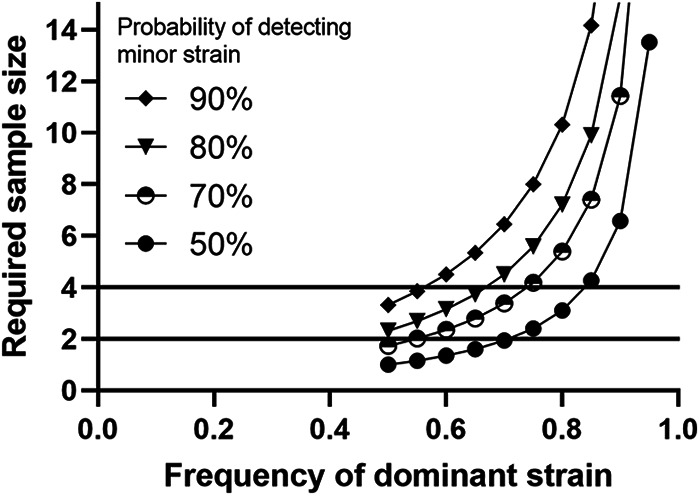
Modeling the required sample size to have a 50% chance or more of detecting the nondominant ST type. Under the assumption of a random sampling of a perfectly mixed population, the outcome would follow a binomial distribution. Derivation of the equation for this scenario can be found in Supplemental File 1.

The low variability of ST in the Legiolert tray is most likely attributed to the low diversity in the sample rather than a bias, considering that when 8 isolates of the composite from another sample from the same building (sample S8-D Table 3) were typed, it was evenly split between these two STs, ST1427 and ST2859. Legiolert has also been reported to detect multiple serogroups in the same samples in another study (23). High ST diversity is also rarely observed in engineered water systems (10, 35, 38, 39) and environmental samples (8). As one strain becomes dominant, the number of isolates that would need to be typed to detect any other strain exponentially increases. Under perfect conditions where the bacterial population can be randomly sampled, the probability of detecting a non-dominant strain can be modeled by a simple binomial distribution (Equation S1-S3). Once the frequency of the dominant strain becomes 0.85 of the population, there is only an ~50% chance of detecting a minor strain if 4 isolates are typed (Fig. 5). For a 90% probability, 14 colonies would need to be typed. Hence, why so many colonies needed to be typed to detect ST1427 in S8-A, where ST2859 represented 91% of isolates.

**FIG 6:**
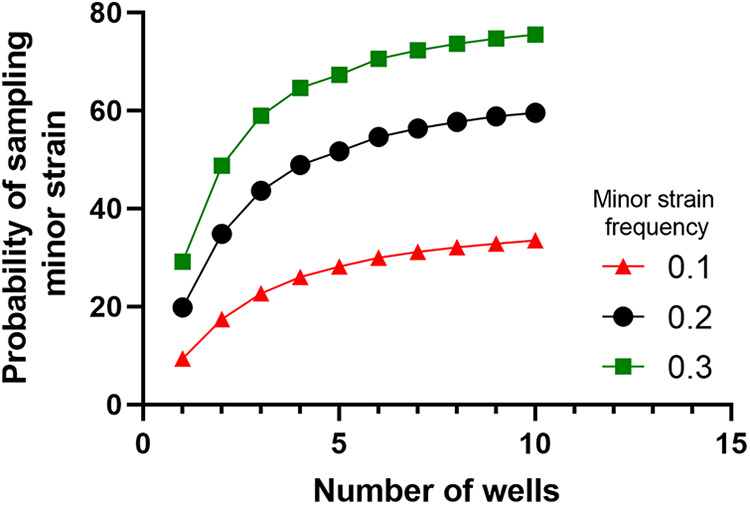
Modeling the optimal number of small wells in a composite to detect the minor strain assuming that (i) the sample was perfectly mixed, (ii) the sample was diluted enough that a single bacteria colonized each small well, (iii) all 96 small wells were positive, and (iv) 5 colonies from composite were screened. The probability of sampling a well containing a minor strain followed a hypergeometric distribution because they were being randomly selected without replacement. The subsequent probability of selecting at least one minor strain colony from that composite would be modeled by the binomial distribution as previously described. Details of the model for this scenario can be found in Supplemental File 1.

A similar type of probability distribution applies when sampling Legiolert wells for making a composite sample. Due to their greater area, large wells are more likely to be inoculated by many bacteria and so the dominant strain will most likely also dominate those wells. Smaller wells capture a smaller number of bacteria, even possibly one, giving minor strains a better chance to overtake them. This is well illustrated by the presence of two variants of ST2859 in the Legiolert tray inoculated with S8-A. In this case, small wells showed only 1 variant, whereas 2 out of the three large wells were able to capture the 2 variants. Therefore, assuming each small well contains a single strain, we can model the probability of selecting a small well that contains the minor strain by a simple hypergeometric distribution because wells are sampled without replacement. The subsequent detection of a minor strain from that composite follows the binomial distribution previously discussed and will change depending on the final proportions of strains in the composite. Combining both and assuming that 5 colonies were screened as recommended in the standard culture protocol, the consequent model calculates the probability of detecting a minor strain given a composite size (Fig. 6). We found that while sampling more wells increases the likelihood of choosing a well colonized by the minor strain, it also decreases the probability of the minor strain representing a significant proportion in the final composite. Hence, there are diminishing returns with increasing composite size, and it begins to plateau after 10 wells. Overall, the composite of 10 wells is at least representative of the dominant strains present in the tray, and likely of those in the original system, and provides a reasonable chance of capturing minor strains. The isolation of the two variants of ST2859 from the composite well supports this conclusion. Legiolert consistently produces many colonies, unlike standard culture which produces few colonies in most cases and so sample sizes can easily be increased if there is interest in detecting minor strains, especially when culturing from large wells or composite.

Access to historical environmental and clinical isolates is incredibly helpful in investigations. For example, Lapierre et al. investigated sources for a large outbreak in New York South Bronx in 2015 with WGS and found that the isolate that caused that outbreak had been present in the Bronx for more than 8 years and had colonized multiple cooling towers during that period (11). However, isolates from environmental samples are not typically stored as only the detection and quantification of L. pneumophila is sufficient for operators in the routine monitoring and maintenance of cooling towers (30). When they are, typically only one colony is preserved from standard culture, and only for a short time, 3 months in Quebec (30). Therefore, L. pneumophila diversity and sources can easily go undiscovered, even with well-organized detection programs (33, 40). The National Legionella Outbreak Detection Program in the Netherlands, which involved the prompt administration of a questionnaire to identify potential sources and a subsequent sampling procedure, only managed to match 41 out of 1991 patients with LD between 2002 and 2012 to an environmental source (40). An outbreak investigation in Montreal in 2019 where historical environmental isolates were limited, coming from only highly contaminated towers, also could not identify a source (33). There is a need to adopt long-term storage strategies for environmental samples.

**FIG 7:**
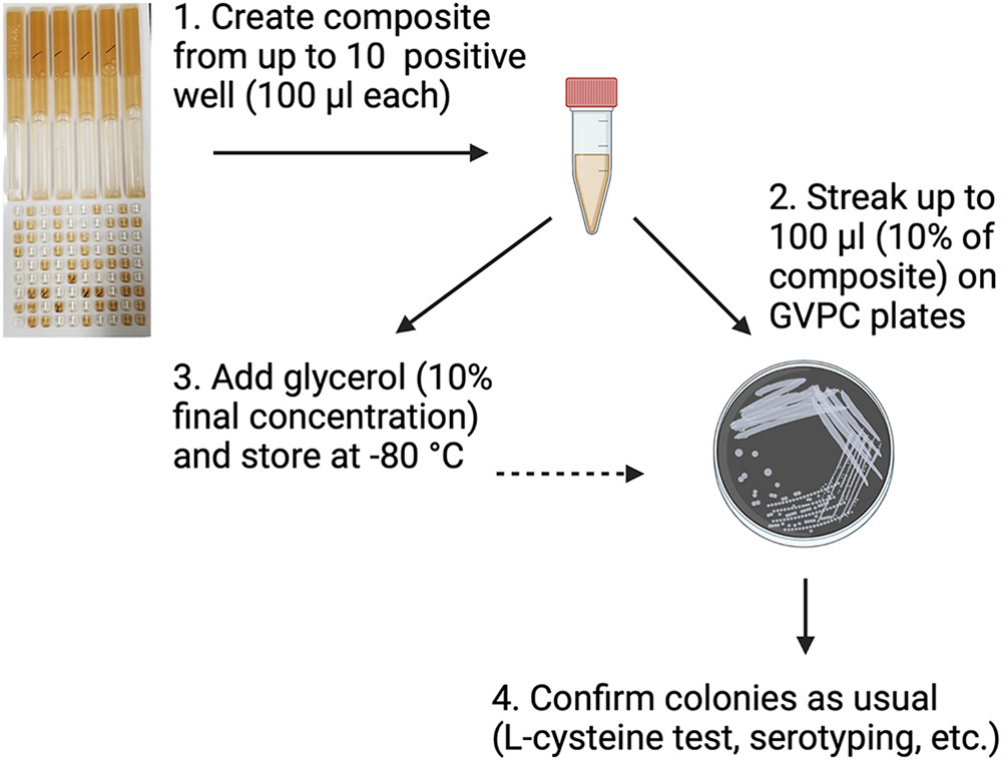
Procedural flowchart for using, storing, and processing Legiolert for L. pneumophila colony isolation. Image created with BioRender.com.

Medium from positive Legiolert wells easily produces hundreds of colonies. Therefore, storing the media directly is equivalent to storing hundreds of isolates and preserving them for future inquiries. This population would include not only the dominant strain but the minor strains as well, which could prove useful during the outbreak investigation. We tested several methods for preserving viable bacteria from Legiolert media by subjecting 100 μL aliquots to different storage conditions and growing them on GVPC plates at multiple time points. Storage of the media at room temperature or freezing directly gave poor results after 2 months (Fig. 2). Storage at 4°C improved longevity to 4 to 6 months but only when the medium was transferred from the tray to a tube. When left in the tray the media slowly evaporates over time, so viability last for less than 4 months (Fig. 1). The addition of 10% glycerol to Legiolert medium extended viability to a minimum of 12.5 months at −20°C, although the number of colonies produced was significantly reduced. When stored at −80°C plates are essentially identical to initial plates 12.5 months before (Fig. 4). For one sample this represented a total of 14.5 months in storage (Fig. 4). This condition would probably allow the preservation of *Legionella* indefinitely. Pellets resuspended in standard storage media, AYE media with 10% glycerol, and stored at −20°C or −80°C were also completely preserved by the end of the storage period. Therefore, the proposed procedure (Fig. 7) for processing and storing Legiolert is to create a composite made of 100 μL from up to 10, preferably small, wells. Multiple composites should be made if there are noted differences in turbidity or color between wells, grouping wells of similar appearance together. Then, 10% or 100 μL, whichever was less, of the composite can be immediately plated on GVPC and incubated at 37°C if isolates are needed at that time, while glycerol is added to a final concentration of 10% to the remainder and stored at −80°C to preserve a population of bacteria. Stored media can be similarly streaked on GVPC to produce colonies. Once colonies appear, at least 5 are subjected to the l-cysteine test. Those that pass this test are considered L. pneumophila and are available for subsequent characterization methods, such as molecular investigation methods like SBT, and storage.

The monitoring and identification of sources for Legionellosis are vital for public health and outbreak studies. SBT and WGS have emerged as powerful tools with good discriminatory power for source identification but require isolates for adequate utilization. Standard culture does provide isolates, but it is an expensive and labor-intensive technique. Legiolert offers an alternative streamlined process for detecting, quantifying, and obtaining L. pneumophila isolates. We confirmed that all known subspecies can be detected by Legiolert. By performing SBT analysis of 96 isolates from this method, we confirm that Legiolert can detect new (ST3054 and ST3069) and clinically relevant (ST1, ST378, ST1427) strains which, when available, matched STs obtained from standard culture isolates in most cases. Legiolert returned more positive results and picked up more STs than standard culture in paired samples. Composites made from equal volumes of multiple wells created a single representative sample, allowing us to detect two strains from one sample. Also, given the typically high number of colonies produced on plates from Legiolert wells, there is an opportunity to type a far greater number of colonies and increase the likelihood of detecting low-frequency strains. Our study also found that the simple addition of 10% glycerol and storage at −80°C, requiring little space and equipment, can preserve Legiolert samples for 12 months and beyond. Overall, Legiolert is a validated alternative to standard culture for obtaining isolates as it similarly captures a variety of common and new STs as demanded by current regulations. Legiolert medium can also be easily stored long-term in a small format, allowing a greater number of historical samples to be kept which enhances the ability to match environmental and clinical strains in epidemiological studies.

## MATERIALS AND METHODS

### Water sample collection.

Water samples were collected from November 2020 to September 2021 from various locations within Québec, Canada. A volume of 1L was collected in sterile plastic bottles and 1 mL of 10% (vol:vol) sodium thiosulfate was added to neutralize chlorine when free chlorine levels were >0.05 mg/L. Potable water samples were taken from the water distribution system of a large public building and a hospital building at various sampling sites, including hot (first draws and flushed for up to 5 min) taps, showers, and recirculating loops. *Legionella* was cultured by Legiolert (IDEXX, USA) according to manufacturer protocol for 100 mL samples (41). Non-potable water samples were taken from the cooling tower (CT) systems of 17 locations, including a large residential building, large public buildings, an industrial site, and a hospital. Samples were most often taken from the water basin or a designated sampling point in the circuit. *Legionella* was cultured according to Legiolert 1 mL nonpotable water protocol (41). For a subset of these samples, we also used AFNOR NF T90-431 standard culture method (20). Briefly, samples were aliquoted into four 1 mL volumes and each was subjected to a different treatment before 100 or 200 μL was plated onto GVPC, which is BCYE medium (recipe in Supplemental File 1) supplemented with 1g/L α-ketoglutarate, 0.4 g/L l-cysteine, 0.25g/L of iron pyrophosphate, and 2 vials of GVPC selective supplement from Sigma-Aldrich (catalog no. 61025-5VL) per liter. One aliquot was plated directly and after a 1/10 dilution in sterile water. The others were either heat-treated at 50°C for 30 min, acid-treated with 1 mL of a pH 2 solution (0.01 M HCl and 0.19 M KCl) for 5 min at room temperature, or both before being plated. Negative (sterile water) and positive (pure L. pneumophila) controls were processed in parallel. All plates were incubated at 37°C for 8 to 11 days. Colonies with *Legionella* morphology were enumerated, and at least five were replated on BCYE with and without cysteine. Those that did not grow in the absence of cysteine were presumed to be *Legionella.* Isolates were confirmed as L. pneumophila after PCR for SBT typing or antibody latex test (Oxoid).

### Verifying detection of Legionella subspecies by Legiolert.

Isolates of *Legionella* subspecies Raphaeli D7705 and Pascullei D1760 were received from the CDC (42). Strains were grown from glycerol stocks on BCYE, then single colonies were inoculated in 5 mL AYE and grown overnight at 37°C with shaking until post exponential phase. Recipes for mediums are in Supplemental File 1. The following day, cultures were centrifuged at 3000 × *g* for 10 min and the pellets were washed twice with sterile Fraquil (43), a defined freshwater medium, before being resuspended in 5 mL of Fraquil and starved at room temperature for at least 3 days. The suspensions were diluted 1/100 four times to obtain a final concentration of 10^3^ to 10^4^ cells/L, confirmed via CFU counts on BCYE. That final suspension was used in the 10 mL potable water protocol for Legiolert and the results were recorded.

### Culturing Legionella from Legiolert trays.

Positive trays were stored at 4°C for up to 1 month before processing. Results were recorded, and up to 10 wells were randomly chosen to create a composite. In the case of highly positive trays, 4 large and 6 small wells were selected to represent both well sizes in the composite. Then, the trays were moved to a biological safety cabinet and decontaminated with 70% ethanol, paying particular attention to the paper backing. Media was removed from selected wells with a needle and syringe from the paper side, and a composite was made by mixing 100 μL from each positive well. In a preliminary experiment, a 100 μL aliquot of the composite was streaked in a quadrant pattern on BCYE, commercial BCYE supplemented with GVPC (Hardy Diagnostics), and in-house BCYE supplemented with GVPC plates made as described in the previous section. Plates were incubated at 37°C for 2 to 3 days until colonies showing typical *Legionella* morphology could be seen. Growth was comparable on all plates (Fig. S1 in Supplemental File 1) but given the higher likelihood of interfering bacteria in nonpotable water samples, in-house GVPC plates were used in all subsequent isolations. Colonies with *Legionella* morphology were then screened by streaking on BCYE plates with and without l-cysteine. A positive control, L. pneumophila ATCC 33152, and negative control, Pseudomonas alcaliphila (JCM 10630), were streaked in the top quadrant of the plate. Colonies that could not grow in the absence of l-cysteine were presumed to be L. pneumophila and were stored individually in AYE media with 10% glycerol at −80°C. For one sample, in addition to the composite, another 9 wells were streaked individually to investigate interwell variability.

### Sequence-based typing.

The isolates described in this study were typed by sequencing seven genes following PCR amplification according to ESGLI protocol (13). Briefly, the DNA template was obtained by emulsifying colonies in 25 μL of 0.1N NaOH and incubating at room temperature for 20 min. Then, 25 μL of 1 M Tris pH 7.4 and 450 μL of sterile Milli-Q were added. Amplification was performed by PCR in a final volume of 50 μL with 1× OneTaq standard reaction buffer, 0.2 μM each primer (Table S1 in Supplemental File 1), 200 μM dNTPs, 1.25 units of OneTaq DNA-polymerase (NEB) and 2 μL of the template. DNA extracted from L. pneumophila strain Philadelphia-1 (ATCC 33152) and 2 μL of sterile PCR-grade water were used as a positive and negative control, respectively, in all PCR amplifications. Tubes were placed in preheated Veriti 96-well thermal cycler (Applied Biosystems) and subjected to the following conditions: initial denaturation at 95°C for 30 sec, 30 cycles of denaturation at 95°C for 15 sec, annealing at 55°C for 15 sec, elongation at 68°C for 1 min, and final elongation at 68°C for 5 min. Products were analyzed by gel electrophoresis in 0.8% agarose gel with ethidium bromide (0.6 μg/mL) and visualized under UV light in the E-Box gel documentation system (Vilber). Successful reactions were purified with Mag-Bind TotalPure NGS (Omega Bio-Tek) and quantified with Quant-iT PicoGreen dsDNA assay kit (Thermo Fisher) according to the manufacturer’s instructions. Concentrations were adjusted to 2 ng/μL and a 30 μL aliquot of purified products and relevant primers for forward and reverse sequencing were sent to the Institute of Integrative Biology and Systems (Université de Laval) for Sanger sequencing using BigDye 3.1 terminators and capillary electrophoresis with ABI 3730xl Data Analyzer (Applied Biosystems). Trace files were compared to those in the European Society of Clinical Microbiology and Infectious Diseases (ESCMID) Study Group for Legionella Infections database using the Benchling platform (https://www.benchling.com/) to determine ST. New allelic profiles were submitted to the ESGLI SBT database.

### Whole-genome sequencing.

The sequence type of all the isolates from site S8 was deduced from whole-genome sequencing. Genomic DNA (gDNA) was extracted from pure isolate cultures using the Wizard Genomic DNA purification kit (Promega) following the manufacturer’s instructions. Quantification, quality check, and sequencing were performed at McGill Applied Genomics Innovation Core. Library preparation was performed with AmpFREE Low DNA Fragment Library kit and sequenced using the Illumina V2 flow cells and 150 bp paired-end reads with the Illumina MiSeq System. Paired-end reads were cleaned with fastp (44) and assembled with spades (45). *In silico* typing was performed using the legsta version 0.5.1 (16). This was successful in typing the ST1427 genome. However, for some isolates typing was hampered by the multiple copies of the *mompS* locus with reads corresponding to *mompS* 7 or 15 occurring at the same frequency. Reads were too short to be resolved *in silico* using Gordon et al. (46) read-filtering strategy based on reading alignments to unique ‘anchors’ sequences in mompS2. Therefore, the *mompS* gene was amplified and sequenced according to the protocol previously outlined. The genomes were queried against multiple databases with the Microbial Genomes Atlas (MiGA) for taxonomic classification into one of the three *Legionella* subspecies *pneumophila*, *fraseri*, and *pascullei* (47). Finally, kSNP version 3.021 was used to perform core single nucleotide polymorphism (SNPs) phylogenetic analysis (48). kSNP was run with a kmer size of 19, as determined using Kchooser. Parsimony trees were visualized and annotated with FigTree version 1.4.4.

### Storage tests.

Storage tests were undertaken to determine which conditions preserved viable and culturable bacteria from Legiolert for at least 12 months. Six trays with at least 2 large positive wells from location S8 (Table 4) were selected. Media from one large well (~15 mL) was removed and 100 μL was used immediately for culture following the procedure described above. The remainder was divided into 100 μL aliquots and subjected to one of the following storage conditions:

1. Stored at 4°C.
2. Stored at room temperature (~21°C).
3. Stored at −20°C.
4. Added glycerol to a final concentration of 10% and stored at −20°C.
5. Added glycerol to a final concentration of 10% and stored at −80°C.
6. Pelleted and resuspended in 10% glycerol in AYE at −20°C.
7. Pelleted and resuspended in 10% glycerol in AYE at −80°C.

The trays, now with one remaining positive well, were stored at 4°C. After 1, 2, 4, 6, and 12 months, one tray was stored at 4°C and 100 μL from each condition, and each replicate was cultured on GVPC plates and incubated at 37°C for 3 days. Images were taken to record the results.

### Data availability.

Sequences for novel SBT have been submitted to the ESGLI SBT database. Access can be requested via email from a professional organization at legionella-sbt@phe.gov.uk. The raw reads for whole-genome sequencing were deposited in a Sequence Read Archive (SRA) with the BioProject accession number PRJNA809832.
