# Correction for Matthews et al., “Detection of Diverse Sequence Types of Legionella pneumophila by Legiolert Enzymatic-Based Assay and the Development of a Long-Term Storage Protocol”

**DOI:** 10.1128/spectrum.00452-23

**Published:** 2023-03-21

**Authors:** Sara Matthews, Hana Trigui, Marianne Grimard-Conea, Elliston Vallarino Reyes, Gabriel Villiard, Dominique Charron, Emilie Bédard, Sebastien P. Faucher, Michèle Prevost

## AUTHOR CORRECTION

Volume 10, no. 6, e02118-22, 2022, https://doi.org/10.1128/spectrum.02118-22. Page 14, Acknowledgments, line 2 from bottom: “d’Immobilisation, Bioalert” should read “d’Immobilisation, Direction générale de la coordination scientifique et du Centre d’expertise en analyse environnementale du Québec, du ministère de l’Environnement, de la Lutte contre les changements climatiques, de la Faune et des Parcs, Bioalert.”

