# Supplementary material for: Detection of Diverse Sequence Types of Legionella pneumophila by Legiolert Enzymatic-Based Assay and the Development of a Long-Term Storage Protocol
Source: Microbiol Spectr. 2022 Oct 31;10(6):e02118-22. doi: 10.1128/spectrum.02118-22 (PMC9769756; doi:10.1128/spectrum.02118-22)
Supplement: Supplemental file 1 — Supplemental material. Download spectrum.02118-22-s0001.pdf, PDF file, 2.1 MB [file spectrum.02118-22-s0001.pdf]

Supplementary Materials

Detection of diverse sequence types of *Legionella pneumophila* by Legiolert enzymatic based assay and the development of a long-term storage protocol

Sara Matthews[1], Hana Trigui[2], Marianne Grimard-Conea[2], Elliston Vallarino Reyes[1], Gabriel Villiard[1], Dominique Charron[2], Émilie Bédard[2], Sébastien Faucher[1,3*] and Michèle Prevost[2,4,*]

1. Department of Natural Resource Sciences, McGill University, Sainte-Anne-de-Bellevue, Québec, Canada, H9X 3V9
2. Department of Civil, Geological and Mining Engineering, Polytechnique de Montréal, Montréal, QC H3C 3A7, Canada
3. Centre de Recherche en Infectiologie Porcine et Avicole (CRIPA), Université de Montréal, Faculté de Médecine Vétérinaire, Saint-Hyacinthe, QC, Canada
4. Industrial Chair on Drinking Water, Department of Civil, Geological and Mining Engineering, Polytechnique de Montréal, Montréal, Quebec, Canada

* Corresponding author: sebastien.faucher2@mcgill.ca or michele.prevost@polymtl.ca

Supplementary Methods

Recipes for media and agar

Buffered-charcoal yeast extract (BCYE) agar - Add 10 g of yeast extract, 10 g of ACES buffer, and 1g of α-ketoglutaric acid potassium salt to 950 mL of de-ionized water. Then adjust the pH to 6.90 +/- 0.05 using 10M KOH. Add 2 g of charcoal and 15 g of agar and autoclave at 121ºC. Cool to 55ºC and aseptically add 10 mL of 4% (w/v) L-cysteine, 10 mL of 2.5% (w/v) of iron pyrophosphate and any other additional supplements before pouring plates.

Charcoal yeast extract (CYE) agar - Add 10 g of yeast extract and 10 g of ACES buffer to 950 mL of de-ionized water. Then adjust the pH to 6.90 +/- 0.05 using 10M KOH. Add 2 g of charcoal and 15 g of agar and autoclave at 121ºC. Cool to 55ºC and aseptically add 10 mL of 4% (w/v) L-cysteine, 10 mL of 2.5% (w/v) of iron pyrophosphate and any other additional supplements before pouring plates.

ACES buffered yeast extract (AYE) liquid - Add 10 g of yeast extract and 10 g of ACES buffer to 950 mL of de-ionized water. Then adjust the pH to 6.90 +/- 0.05 using 10M KOH. Autoclave at 121ºC. Cool to 55ºC and aseptically add 10 mL of 4% (w/v) L-cysteine, 10 mL of 2.5% (w/v) of iron pyrophosphate and any other additional supplements before pouring plates.

Sampling model using binomial distribution

The binomial distribution formula can be used to calculate the probability of 'x' outcomes in 'n' independent trials, given the probability 'p' of 'x' for each trial as follows:

Equation S1: $P_{(x)} = \left(\frac{n!}{(n-x)!x!}\right) p^x q^{n-x}$

Where $P_{(x)}$ is the binomial probability, x is the number of times for a specific outcome, n in the number of trials, p is the probability of success in a single trial and q, the probability of failure. In applying it to sampling *Legionella* colonies, the probability of detecting any ST type is the same as its frequency in the population. Given the large population size, we can assume frequency will remain constant between sampling trials. Hence this scenario fulfills the assumptions for a binomial distribution. While there can be more than two STs within a population, we are only concerned with the probability of detecting the non-dominant strain. Therefore, ST can be split into two categories, dominant and non-dominant, and the probability of detecting a non-dominant strain at least once in a set of trials ($P_{(\geq 1)}$) simply becomes the inverse of the probability of detecting only the dominant strain as shown in the equation below:

Equation S2: $P_{(\geq 1)} = 1 - frequency\ of\ dominant\ strain^n$

Re-arranging to isolate for n:

Equation S3: $n = \frac{\log(1 - P_{(\geq 1)})}{\log\,(frequency\ of\ dominant\ strain)}$

Figure 5 was created by fixing $P_{(\geq 1)}$ and varying the frequency of the dominant strain.

Well sampling model using hypergeometric distribution

The hypergeometric distribution can be used to calculate the probability of obtaining ‘k’ outcomes in ‘n’ trials given that we are randomly sampling, without replacement, from a population size ‘N’ as follows:

Equation S3: $P(X = k) = \frac{\binom{K}{k}\binom{N-K}{n-k}}{\binom{N}{n}}$

Where K is the number of successes in the population, k is the number of observed successes, N is the population size, n is the number of draws, inserted in the combination formula:

Equation S4: $\binom{a}{b} = \frac{a!}{b!(a-b)!}$

Here it can be applied to determine the chance of selecting a certain number of wells with the minor strain given that 1) samples were diluted enough that a single bacteria inoculated each small well 2) the proportion of wells containing the minor strain is the same as its frequency in the sample 3) all 96 small wells are positive. Using a minor strain frequency of 0.2 as an example we can generate the following set of probabilities for different composite sizes in the table below:

		Probability of selecting ‘k’ wells with the minor strain in composite										
Number of wells sampled	Number of minor strain wells	0	1	2	3	4	5	6	7	8	9	10
1		0.802	0.198									
2		0.642	0.321	0.038								
3		0.512	0.389	0.092	0.007							
4		0.407	0.418	0.151	0.022	0.001						
5		0.323	0.421	0.205	0.046	0.005	1.90E-04					
6		0.256	0.405	0.250	0.076	0.012	9.66E-04	2.93E-05				
7		0.202	0.378	0.283	0.110	0.024	0.003	1.75E-04	4.23E-06			
8		0.159	0.345	0.306	0.144	0.040	0.006	5.99E-04	2.93E-05	5.70E-07		
9		0.124	0.308	0.317	0.177	0.059	0.012	0.002	1.14E-04	4.49E-06	7.12E-08	
10		0.097	0.272	0.319	0.207	0.081	0.020	0.003	3.27E-04	1.96E-05	6.31E-07	8.19E-09

Each possibility above results in a different proportion of the minor strain in the final composite and will affect the probability of sampling the minor strain from that final composite as in equation S2. Assuming 5 colonies will be screened from the composite and applying equation S2, the following probabilities are calculated for each case:

		Probability of selecting at least one minor strain colony (n =5) given certain proportion in composite										
Number of wells sampled	Number of minor strain wells	0	1	2	3	4	5	6	7	8	9	10
1		0.000	1.000									
2		0.000	0.969	1.000								
3		0.000	0.868	0.996	1.000							
4		0.000	0.763	0.969	0.999	1.000						
5		0.000	0.672	0.898	0.978	0.998	1.000					
6		0.000	0.598	0.868	0.969	0.996	1.000	1.000				
7		0.000	0.537	0.814	0.939	0.986	0.998	1.000	1.000			
8		0.000	0.487	0.763	0.905	0.969	0.993	0.999	1.000	1.000		
9		0.000	0.445	0.715	0.868	0.947	0.983	0.996	0.999	1.000	1.000	
10		0.000	0.410	0.672	0.832	0.922	0.969	0.990	0.998	1.000	1.000	1.000

Multiplying the probabilities together for each case, and suming along the rows gives the final probability of detecting the minor strain for a given composite size:

		Probability of obtaining that composite AND selecting at least one minor strain colony from it (n =5)											
Number of wells sampled	Number of minor strain wells	0	1	2	3	4	5	6	7	8	9	10	Total
1		0.000	0.198										0.198
2		0.000	0.311	0.038									0.348
3		0.000	0.338	0.092	0.007								0.436
4		0.000	0.319	0.146	0.022	0.001							0.489
5		0.000	0.283	0.184	0.045	0.005	0.000						0.517
6		0.000	0.242	0.217	0.074	0.012	0.001	0.000					0.546
7		0.000	0.203	0.231	0.103	0.023	0.003	0.000	0.000				0.564
8		0.000	0.168	0.233	0.131	0.038	0.006	0.001	0.000	0.000			0.577

9		0.000	0.137	0.227	0.154	0.056	0.012	0.002	0.000	0.000	0.000		0.588
10		0.000	0.111	0.214	0.172	0.075	0.020	0.003	0.000	0.000	0.000	0.000	0.596

Figure 6 was created by plotting the total probability against the composite size.

Supplementary Figures

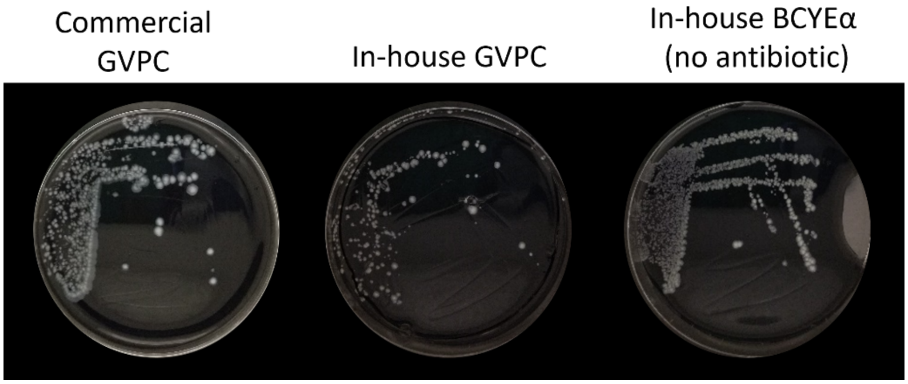

Figure S1 – Comparison of different plates for the isolation of *L. pneumophila* from Legiolert.

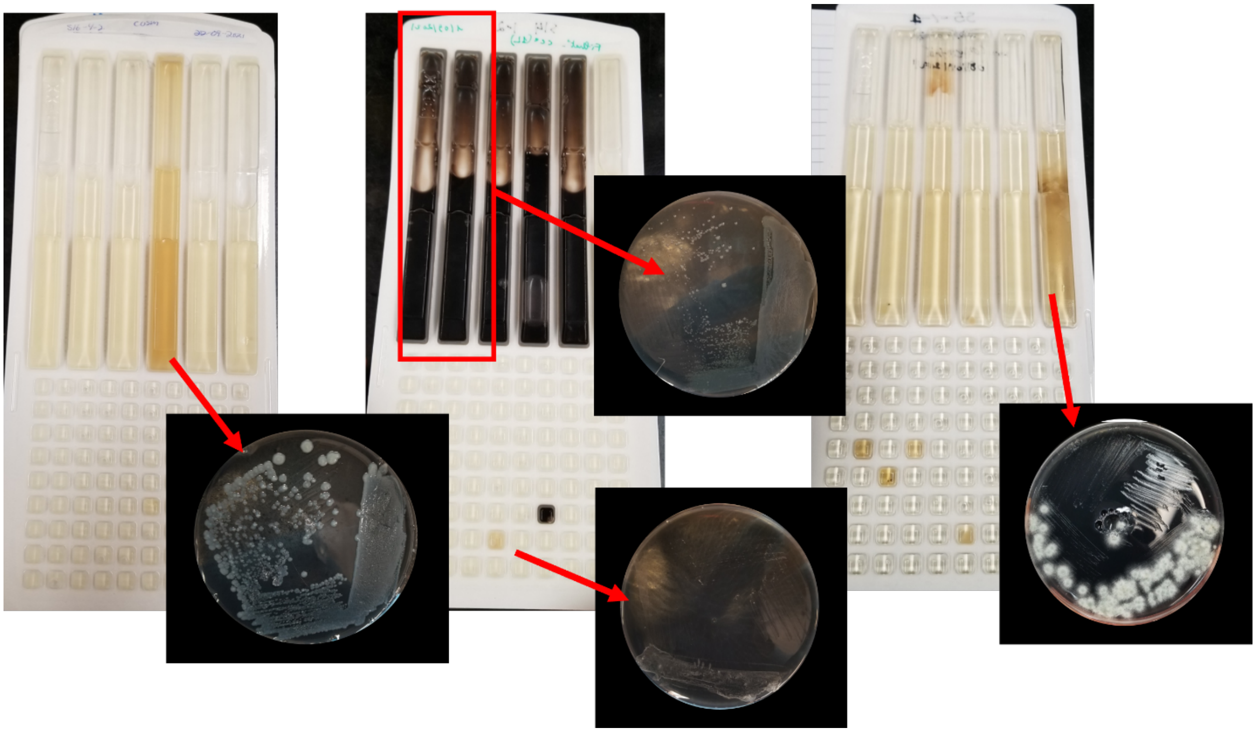

Figure S2 – Legiolert trays with atypical results due to interfering flora. Interfering flora can produce obviously abnormal well appearance such as black wells or floating mold (centre and right). Or it is discovered when non-Legionella colony morphology appears on GVPC (centre and right) plate or failure of subsequent cysteine test (left).

Supplementary Tables

Table S1 – ESGLI primers for PCR amplification and sequencing of genes for sequence-based typing.

Gene	Primer name	Position	Primer sequence (5'-3')	Annealing temperature (°C)	Fragment size (bp)
flaA	flaA-587F<br>flaA-960R	568-587<br>981-960	GCG TAT TGC TCA AAA TAC TG<br>CCA TTA ATC GTT AAG TTG TAG G	55	414
pile	pilE-35F<br>pilE-453R	12-35<br>471-453	CAC AAT CGG ATG GAA CAC AAA CTA<br>GCT GGC GCA CTC GGT ATC T	55	460
Asd	asd-511F<br>asd-1039R	487-511<br>1062-1039	CCC TAA TTG CTC TAC CAT TCA GAT G<br>CGA ATG TTA TCT GCG ACT ATC CAC	55	576
Mip	mip-74F<br>mip-595R	58-74<br>616-595	GCT GCA ACC GAT GCC AC<br>CAT ATG CAA GAC CTG AGG GAA C	55	559
mompS	mompS-450F<br>mompS-1116R	430-450<br>1140-1116	TTG ACC ATG AGT GGG ATT GG<br>TGG ATA AAT TAT CCA GCC GGA CTT C	55	711
proA	proA-1107F<br>proA-1553R	1090-1107<br>1570-1553	GAT CGC CAA TGC AAT TAG<br>ACC ATA ACA TCA AAA GCC	55	481
neuA	neuA-196F<br>neuA-634R	176-196<br>634-611	CCG TTC AAT ATG GGG CTT CAG<br>CGA TGT CGA TGG ATT CAC TAA TAC	55	459

Table S2 – Allele profile of ST identified.

SBT	*flaA*	*pilE*	*asd*	*mip*	*mompS*	*proA*	*neuA*
**1**	1	4	3	1	1	1	1
**378**	3	13	1	17	14	9	7
**1427**	3	12	1	6	14	9	220
**2859**	11	14	16	12	15	13	9
**3054**	3	10	1	6	1	9	1
**3069**	7	10	17	6	17	11	9
